# Integration of Routine Parameters of Glycemic Variability in a Simple Screening Method for Partial Remission in Children with Type 1 Diabetes

**DOI:** 10.1155/2018/5936360

**Published:** 2018-01-17

**Authors:** Nina Nielens, Olivier Pollé, Annie Robert, Philippe A. Lysy

**Affiliations:** ^1^Pediatric Endocrinology Unit, Cliniques Universitaires Saint Luc, Av. Hippocrate 10, 1200 Brussels, Belgium; ^2^Pôle Epidémiologie et Biostatistique, Institut de Recherche Expérimentale et Clinique, Université Catholique de Louvain, Av. Hippocrate 10, 1200 Brussels, Belgium

## Abstract

Although different criteria were used to define partial remission in type 1 diabetes, the IDAA1C formula has prevailed as it correlates with stimulated C-peptide levels. Our retrospective study evaluated clinical variables associated with the occurrence of IDAA1C-defined partial remission in a series of 239 pediatric patients. Diabetic ketoacidosis and age at diagnosis, but no other clinical feature, influenced the occurrence of remission. We then evaluated whether parameters of glycemic variability used in clinical routine may reliably define partial remission, as these would alleviate confounding factors related to insulin treatment. Using multiple linear regression, we observed that HbA_1C_ levels and percentage of normoglycemia were efficient and sufficient to predict partial remission. These parameters were entered into a formula, called glycemic target-adjusted HbA_1C_ (GTAA_1C_), that corresponded to HbA_1C(%)_ − (3 × % of normoglycemic values_(70–180 mg/dL)_). With a threshold of 4.5, this alternative formula predicted partial remission with a sensitivity and a specificity of 72.3% and 92%, respectively, and yielded strong correlation with IDAA1C levels and BETA-2 score, which is a correlate of *β*-cell function after islet transplantation. We propose GTAA_1C_, based on routine and objective markers of glycemic variability, as a valid alternative for definition of partial remission in type 1 diabetes.

## 1. Introduction

In type 1 diabetes (T1D), there is a longstanding autoimmune attack of pancreatic *β*-cells [1] recognizable by seroconversion of specific antibodies [2] that develops on genetic susceptibility grounds [3] and leads to symptomatic insulinopenia when *β*-cell mass is drastically reduced [4]. Since the fall of insulin stores is abrupt, it is thought that dysregulation of glucose homeostasis is contemporaneous to overt onset (i.e., polyuria and polydipsia) of the disease. Alleviation of hyperglycemia by administration of exogenous insulin is accompanied in about 60% of patients by a rapid reduction of daily insulin requirements (DIR) for maintenance of normal glycemia and HbA_1C_ levels [5]. This defines a transitory state of partial remission (PR) (or “honeymoon period”) with residual *β*-cell function, improved insulin sensitivity [6], and reduced risk of severe hypoglycemia (SH) [7, 8]. As such, PR represents a key period—between 7 and 9 months [9, 10]—in the early management of diabetes: PR seems to be optimal to introduce new diets, immunotherapies, and strategies to preserve and/or expand *β*-cell mass [4, 11].

The definition of PR, being of particular clinical importance, has been variously addressed and remains a matter of debate [12]. The Hvidoere study group on childhood diabetes proposed the identification of remitters using the insulin dose-adjusted hemoglobin A_1C_ (IDAA1C) formula [13], which strongly correlated with residual *β*-cell function estimated by stimulated C-peptide levels during mixed-meal tolerance test, when being lower or equal to 9. To validate the IDAA1C definition, the Hvidoere cohort was further compared to a Danish cohort of patients, which had different age and C-peptide secretion profiles, such that the sensitivity and specificity of IDAA1C to predict C-peptide levels were lower than expected [14]. Similarly, Hao et al. described good correlations between IDAA1C and peak C-peptide (>0.2 pmol/mL) levels during the first three years after diagnosis, but rather low sensitivity of IDAA1C itself (≈50% in children and ≈67% in adults) [15]. Yet the IDAA1C threshold (i.e., ≤9) was successfully used elsewhere to identify remitters [10, 16, 17] and level out other parameters, such as daily insulin dose per kilogram of body weight [12].

A common feature of clinically meaningful PR is that patients harbor low levels of glycemic variability (GV) (e.g., standard deviation, coefficient of variability, and percentage of normoglycemia), which is a recognized feature of residual *β*-cell function since more than three decades [18]. As opposed to daily insulin dose, parameters of GV might per se represent a better assessment of PR since it only refers to objective measures, whereas for patients without electronic logs of insulin doses, correction units may not always be recorded [19]. In this study, we analyzed a retrospective cohort of patients with the aim to develop a definition of PR using parameters independent of DIR and which significantly correlates with hallmarks of *β*-cell function.

## 2. Patients and Methods

The study was designed as an observational study with a retrospective cohort of 239 children and adolescents with T1D-attending outpatient clinic in a tertiary health care center (Cliniques Universitaires Saint Luc) and followed in our pediatric diabetes clinic from diagnosis (from 1998 to 2013) to adulthood (18–20 years of age). The local ethical committee approved the study protocol. The study was conducted in accordance to the Declaration of Helsinki. T1D was diagnosed according to International Society for Pediatric and Adolescent Diabetes (ISPAD) guidelines [20] and based on symptoms of insulinopenia, elevated blood glucose (expressed in mg/dL) and HbA_1C_, positive anti-islet antibodies (GAD65, IA2, and insulin), and lack of family history of genetic diabetes. Biometrics (age, height z-score, and BMI z-score) and biological features (blood glucose, HbA_1C_) were collected at diagnosis and at each consultation (postdiagnosis consultations occurred at 15 days, 1 month and then every 3 months; only fully adherent patients were recorded). At diagnosis, measures included screening of DKA (defined as pH < 7.3 and/or bicarbonate < 16 mM) and postprandial C-peptide levels (AutoDELFIA C-peptide, PerkinElmer Life and Analytical Sciences), which were assayed every year. Z-scores for height and BMI were assessed using Belgian Flemish reference charts [21]. HbA_1C_ was determined by high-capacity liquid chromatography with iron-resin exchange.

Insulin doses were adjusted for pre- and postprandial glycemic targets according to ISPAD guidelines [20], when available, or to our institution's guidelines. SH was defined as loss of consciousness, coma with or without convulsions, or alteration of consciousness impeding the capacity for oral sugar ingestion (need of a tier for IM glucagon administration). Occurrence of SH was monitored at each consultation (as per our institution's guidelines). Only patients that performed at least five measurements of capillary BG were included in the study. Self-monitoring data were recorded during each consultation. PR was defined as IDAA1C ≤ 9, according to definition by Mortensen et al. [13]: A1C (%) + [4 × insulin dose (U/kg/day)].

Data were analyzed using the GraphPad and Sigmaplot software. Categorical variables were analyzed using chi-square test or Fisher's exact test for small samples. Continuous variables were analyzed using unpaired *t*-test or Mann–Whitney rank sum test, according to the statistical distribution. ANOVA with or without R tests was used, according to the statistical distribution, when there were more than two groups. Normality of distribution was verified through Shapiro-Wilk testing. For continuous variables, data were expressed as mean ± standard deviation when normally distributed, and as median and interquartiles (q25%–q75%) when not. Correlation analysis was used to evaluate relationship between variables. When building logistic regression models, all significant variables in univariate analyses were entered into a multivariate logistic regression. Results are expressed as odds ratio (OR) with 95% confidence intervals. Logistic regression analyses were performed using IBM SPSS Statistics 21.0 software. *P* < 0.05 was considered significant.

## 3. Results

### 3.1. Determinants of Partial Remission

In the 239 newly diagnosed patients with T1D, remission occurred in 71.1% (*n* = 170, all being partial) with similar rates of remission in girls (46.9%) and boys (53.1%), although girls were significantly older than boys at diagnosis (Table 1). While age at diagnosis did not influence PR occurrence globally, children less than 5 years of age were significantly less likely than children aged 5–10 years to enter PR (resp., 59% and 77.3%, *P* = 0.035). When children were grouped according to gender, a striking age stratification of PR risk was found for girls only: no more than 19.2% of girls diagnosed before the age of 5 entered PR as compared to 78% and 91% in the groups aged 5–10 and ≥10 years old, respectively (*P* < 0.001). Mean duration of PR was 8.9 ± 8.6 months (range 1.8–44.3), without influence of gender or age.

**Figure 1:**
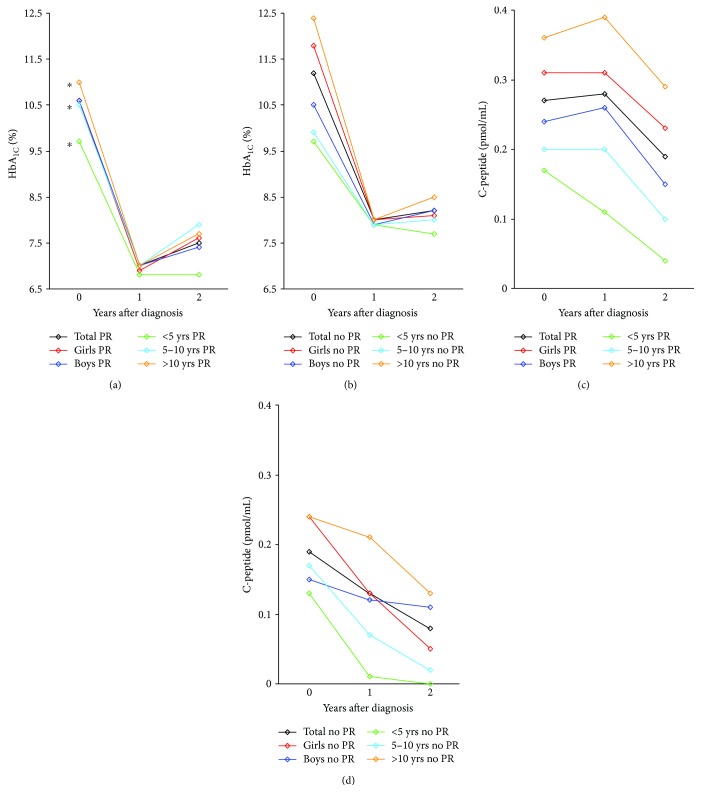
Evolution of HbA_1C_ and C-peptide values at diagnosis and during follow-up. Graphs represent mean HbA_1C_ levels (in %) in PR (a) and no PR (b) groups, mean C-peptide values (in pmol/mL) in PR (c) and no PR (d) groups at diagnosis, and one and two years postdiagnosis. Mean HbA_1C_ levels were at 10.6 ± 2.6% in PR and 11.2 ± 3% in no PR group (*P* = 0.33). Those levels were, respectively, at 6.9% (6.2–7.5) and 7.7% (6.9–8.6) at one year (*P* < 0.001) and at 7.5% (6.7–8.1) and 7.7% (6.9–8.5) at two years (*P* = 0.023), in patients with PR and without PR. For the remitter group, median C-peptide levels were, respectively, at 0.21 pmol/mL (0.11–0.35), 0.22 pmol/mL (0.1–0.41), and 0.11 pmol/mL (0–0.28) at diagnosis, one year and two years postdiagnosis. For the nonremitter group, median C-peptide levels were, respectively, at 0.15 pmol/mL (0.1–0.23), 0.05 pmol/mL (0–0.17), and 0 pmol/mL (0–0.09) at diagnosis, one year and two years postdiagnosis. ^∗^Compared HbA_1C_ levels at diagnosis among age subgroups (i.e., 9.7 ± 1.9%, 10.4 ± 2.2%, and 11.4 ± 3.1% for the <5 years, 5–10 years, and ≥10 years, resp.; *P* = 0.0017).

Characteristics of DKA at diagnosis and of HbA_1C_ and C-peptide evolution are described in Table S1 and Figure 1. Data were similar to what we described earlier [10], with few exceptions. At baseline, mean HbA_1C_ levels were 10.8 ± 2.7% and positively correlated with age, but not with gender, in the PR group (HbA_1C_ of 9.7 ± 1.9%, 10.4 ± 2.2%, and 11.4 ± 3.1% for the <5 years, 5–10 years, and ≥10 years, resp.; *P* = 0.002) (Figures 1(a) and 1(b)). Median basal C-peptide levels at diagnosis and after one and two years were 0.19 (0.1–0.33) pmol/mL, 0.16 (0.05–0.35) pmol/mL, and 0.08 (0–0.22) pmol/mL, respectively (*P* < 0.001) (Figures 1(c) and 1(d)). At baseline and during follow-up, children >10 years of age had significantly higher C-peptide levels than other age groups, as described elsewhere [10, 14]. Also, C-peptide values at baseline were higher in girls (0.21 [0.12–0.39] pmol/mL) than in boys (0.16 [0.07–0.30] pmol/mL) (*P* < 0.01) but this difference was not observed later on during follow-up. In our cohort, the presence of PR according to the IDAA1C definition was predictive of a C-peptide value higher than 0.3 pmol/mL, with a 51% sensitivity and 80.2% specificity. The best correlation was observed among children <5 years at diagnosis, with a sensitivity of 57.7% and a specificity of 84.8%. In multivariate logistic regression, DKA (*P* = 0.04) and an age of 5–10 years (*P* = 0.01) were the only variables at diagnosis that were associated with a higher chance of experiencing PR.

We found 87.5% and 71.7% of patients positive at diagnosis for anti-IA2 and anti-GAD65 antibodies, respectively. Girls were more likely to be positive for anti-GAD65 than boys (84% versus 60.7%, resp.; *P* = 0.002). When comparing patient subgroups, no significant difference was found for islet antibody titers (i.e., anti-IA2, anti-GAD65, and anti-insulin) and no association could be found between these titers and PR occurrence. Positive (i.e., ≥20 U/mL) anti-transglutaminase antibodies were found in 4.6% of patients at diagnosis, without correlation with the onset of PR. Also, during the three-year follow-up after diagnosis, only a trend toward lower risk of SH episodes in the PR group could be observed (23.5% in PR versus 34.8% in no PR group, resp.; *P* = 0.075) (Table S2). However, this difference was significant when we considered only patients aged <10 years (29.7% in PR versus 55.6% in no PR group; *P* = 0.006). Finally, we found no seasonal influence on the probability of PR.

### 3.2. Prediction of PR Based on Routine Parameters of GV

We aimed to predict PR with indexes of glucose homeostasis used in clinical routine, that is, HbA_1C_, percentage of normoglycemia (% normoglycemia), mean blood glucose, standard deviation to the mean (SD), and coefficient of variation (CV, equal to DS divided by mean blood glucose). When these parameters were run in multivariate analysis, we observed that only HbA_1C_ levels and percentage of normoglycemia significantly (with a significant *P* value or with a significant correlation coefficient) influenced PR prediction as defined by the IDAA1C criterion (Table S3). These were integrated into a new formula for PR prediction, as follows: glycemic target-adjusted HbA_1C_ or GTAA_1C_, being equal to HbA_1C(%)_ − (3 × [% normoglycemia_(70–180 mg/dL)_]), predicted PR when scored ≤4.5. As expected, GTAA_1C_ strongly correlated with IDAA1C (*r* ^2^ = 0.71, *P* < 0.001) and predicted IDAA1C-defined PR with 73.2% sensitivity and 92% specificity. When GTAA_1C_ was evaluated for its capacity to predict PR in patients from different age groups, it showed high specificity for patients <5 years (99.3%, with 64.4% sensitivity, *r* ^2^ = 0.79) and high sensitivity for patients >10 years (80.9%, with 86.5% specificity, *r* ^2^ = 0.73), whereas for patients aged 5–10 years, sensitivity and specificity 67.7% and 95.4%, respectively, (*r* ^2^ = 0.75). No effect of gender was found on sensitivity and specificity of GTAA_1C_.

With GTAA_1C_, PR rates were slightly lower than with IDAA1C: 66.1% of patients entered PR, among those 70.5% of girls and 62.2% of boys were included (*P* = 0.17). Interestingly, there was a significant age-dependent distribution of PR rate with the GTAA_1C_ definition, as PR occurred in 43.6%, 60.2%, and 78.6%, respectively, in children aged <5 years, 5–10 years, and ≥10 years at diagnosis (*P* < 0.001). Also, GTAA_1C_-defined PR were slightly shorter than its IDAA1C counterpart and averaged 8.3 ± 8.04 months without differences for gender or age.

Levels of GTAA_1C_ at 3 months postdiagnosis were inversely correlated with PR duration (*r* ^2^ = 0.26, *P* < 0.001) and, when calculated to be ≤3.5, between 3.5 and 4 and between 4 and 4.5, these levels predicted a PR duration >300 days in 47.9%, 29.3%, and 5.9% of patients (*P* < 0.001) (Figure S1). Among children with GTAA_1C_ values at 3 months postdiagnosis >4.5, only 22% experienced PR, whereas when it levelled above 5, only 11.7% of patients had characteristics of PR. These predictions were not significantly different when PR was characterized according to the IDAA1C criterion (Figure S1).

**Figure 2:**
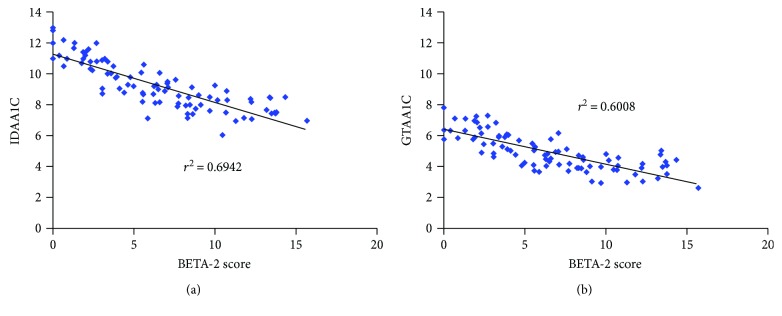
Correlation of BETA-2 score with IDAA1C and GTAA_1C_ definitions of PR. Graphs show correlation between BETA-2 score and IDAA1C-based ((a) *P* < 0.001) or GTAA_1C_-based ((b) *P* < 0.001) criteria for PR in a subgroup of 90 patients from our cohort. These correlations (BETA-2 and IDAA1C versus BETA-2 and GTAA_1C_) were not statistically different in multivariate analysis. Related *r* ^2^ were noted in the corresponding graphs.

Because the GTAA_1C_ definition only refers to parameters of GV, we confronted its levels calculated in our series of patients with a score reflecting *β*-cell function. We chose the BETA-2 score [22], which integrates biomarkers extracted from a morning fasted blood sample (i.e., C-peptide [nM], plasma glucose [mM], HbA_1C_ [%]) and DIR (total units/kg body weight) and which was validated in the setting of islet transplantation in patients with T1D. Because we wanted to limit our investigations in patients with a regular follow-up, by excluding all samplings performed in a clinical trial setting, we restricted our analysis to patients with blood samples drawn during the consultation, that is, in postprandial state. Comparison of BETA-2 score with both IDAA1C and GTAA_1C_ yielded robust correlation criteria (*r* ^2^ = 0.69 and 0.60, resp.) (Figure 2). This confirmed, at least indirectly, the potential of GTAA_1C_ to reflect residual *β*-cell function in patients with T1D.

## 4. Discussion

Although several groups studied characteristics of PR in patients with T1D [9, 13, 23, 24], since 2009, most common prediction studies relied on the IDAA1C formula, which is a valid predictor of PR and stimulated C-peptide values above 0.3 pmol/mL [13]. Despite being validated by different authors [10, 14, 15, 25] and widely used [10, 11, 26, 27], IDAAC1 has two main limitations. First, the correlation of IDAA1C with C-peptide loses specificity and sensitivity with age [13, 14, 17] and tends to underestimate C-peptide levels in children presenting a score above 9 [14–16]. This is especially true in the “>10-year” group where this formula does not discriminate between residual insulin secretion and increased insulin resistance [13, 14]. Second, this score depends on two variables: HbA_1C_ and DIR, which may depend on multiple confounding factors such as hospital guidelines, clinicians' habits, and patient/parents own management of insulin injections. Moreover, DIR estimation does not take into account insulin correction doses [15, 17]. PR might thus be better defined by objective parameters of GV, as those correlate with glucose control [28].

Hirsch et al. [29] define GV as the degree to which a patient's blood glucose level fluctuates between high (hyperglycemia) and low (hypoglycemia) levels, which are known inducers of oxidative stress [30–33], increased comorbidities, and lower residual C-peptide secretion [4, 28, 34]. GV is per se associated with increased cardiovascular risk, as shown in nondiabetic subjects and in diabetic patients stratified for GV parameters but having similar HbA_1C_ levels [35–37]. Among multiple GV parameters [38], clinicians only use in routine the percentage of normoglycemia, the mean glucose level and related standard deviation, and the coefficient of variation of glucose. In 2015, Buckingham and coworkers [16] showed that the percentage of normoglycemia, although not directly integrating peak and nadir glycemic values, is a good predictor of stimulated C-peptide levels above 0.2 pmol/mL if 60% of glucose measurements lie between 3.9 and 7.8 mM, although a clear-cut threshold value was not met. This study suggests the potential of simple GV parameters to serve as variables in the definition of PR.

In our study, we first investigated prevalence and key indicators of remission in a cohort of children with new-onset T1D and then proposed an alternative formula to IDAA1C to predict PR independently of DIR. Using the IDAA1C formula, we found a prevalence of remission of 71% with a mean PR duration of 8.9 months and a peak prevalence 3 months after onset, as described elsewhere [10, 13, 14, 17, 25] even with different PR definition [39]. Also in agreement with previous studies [9, 10, 27, 40, 41] were the findings that T1D onset in patients between 5–10 years and higher C-peptide levels at diagnosis were more frequent in remitters, that younger onset (<5 years) of T1D was associated with a lower rate of PR and that presence of DKA (pH < 7.3) at diagnosis was negatively correlated with PR. Also, while other studies [10, 17, 42] described a negative correlation between anti-islet antibodies and PR, we found no such correlation within our cohort, which may partly be explained by the retrospective design of our study. We also did not find correlation between gender, HbA_1C_ levels, and season at diagnosis with PR. A negative association between the risk of SH in the two first years postdiagnosis and age at diagnosis was observed, in accordance with other studies [43–46] and might be explained by difficulties to recognize and react to symptoms of hypoglycemia, and to the lack of consistent meals in the youngest group of patients. We also found a significant reduction of SH rates in patients <10 years that entered PR (23.5% versus 34.8%).

After validating IDAA1C in our cohort, we aimed to generate a new formula integrating GV parameters (as suggested elsewhere [16]) that can be easily measured in routine clinical practice. We therefore ran multiple linear regression to compare IDAA1C with different GV variables and isolated two parameters correlating with IDAA1C: HbA_1C_ and percentage of normoglycemia. A new alternative PR definition, independent of DIR and integrating GV parameters, was generated and corresponded to GTAA_1C_ (being equal to HbA_1C(%)_ − [3 × % normoglycemia_(70–180 mg/dL)_]), predictive of PR when ≤4.5. Since GTAA_1C_ was generated by reference to IDAA1C, the correlation between both formula was strong (*r* ^2^ = 0.71). GTAA_1C_ yielded high sensitivity (72.3%) and specificity (92%) in predicting IDAA1C-defined PR. In our patients, prevalence of PR and mean PR duration was slightly lower with GTAA_1C_ (66.1% and 8.3 months) than with IDAAC (71.1% and 8.9 months). Using GTAA_1C_, PR prediction was more specific (99.3%) in younger children and more sensible (80.9%) in older children groups but maintained good correlation scores with IDAA1C throughout every age subgroups. GTAA_1C_ tended to underestimate PR in young children (41% versus 59%) due to lower sensitivity (64.4%) in the young children group (<5 years) and to overestimate PR in older children (>10 years) (75% versus 70.5%) due to lower specificity (86.5%).

Episodes of SH were previously shown to be reduced in patients with T1D and residual-stimulated C-peptide levels >0.04 pmol/mL [8, 34] and in patients with late-onset diabetes [43–46]. In a study reanalyzing DCCT data, Kilpatrick and colleagues [47] demonstrated independent correlation between SH and each three of these parameters, HbA_1C_, mean blood glucose and GV (each of those three being more stable during PR). Therefore, PR should be considered as a protective factor against SH, as we observed in our study for children <10 years (*P* = 0.006). Paradoxically, we found no significant differences in the risk of presenting SH between remitter and nonremitters in the >10-year group, which might be explained by the insufficient discrimination of patients with reduced insulin sensitivity by IDAA1C in the postpubertal group.

C-peptide secretion is considered as the gold standard measure for residual insulin secretion in diabetic patients [48]. Pioneer studies [49, 50] have shown increased C-peptide secretion three months after initiation of insulin therapy, linking stimulated C-peptide with PR occurrence [49]. One limitation of our study comes from C-peptide measurements performed randomly rather than after fasting or stimulation tests. However, our investigations were aimed at characterizing PR and developing new PR definition by using routine clinical parameters. Stimulated C-peptide tests might represent a better reflect of *β*-cell function [51] but lacked prediction value for description of PR [14, 16] or to assess clinically significant endogenous insulin secretion in immunoprevention studies [52] or in longitudinal studies of patients with longstanding T1D [53]. Moreover, our data converged with previous studies that characterized C-peptide values at diagnosis of T1D and during follow-up [13, 15, 16].

*β*-Score [54], revised as BETA-2 score [22], was developed to determine graft function after islet transplantation in patients with complicated T1D. *BETA*-2 score is calculated on biological markers measured in a single blood sampling (i.e., fasting C-peptide, fasting glucose, DIR, and HbA_1C_ levels) and negatively correlates with stimulated glucose values. Parameters of GV are other valuable tools to evaluate outcomes of islet grafts. The team of Vantyghem et al. [55] found that mean glucose was a valid determinant of islet graft function with negative (*R* = −0.65 with *β*-score) correlation with continuous glucose-monitoring values. Furthermore, Barton et al. [56] showed that GV and SH were competitive parameters, as compared to insulin doses, to predict islet secretion function. Recent study showed that GV evaluated by SD glucose and CONGA4 score was more reliable than HbA_1C_ to assess islet function and risk of clinical events such as SH [57]. We therefore compared GTAA_1C_ with BETA-2 score in our cohort of patients and found a strong correlation of *r* ^2^ = 0.60 between those parameters, indirectly confirming GTAA_1C_ ability to reflect residual *β*-cell function.

In this study, we describe characteristics of PR in a Belgian cohort of pediatric patients with T1D and underline the paucity of clinical determinants, besides DKA, for prediction of PR occurrence. Moreover, we propose the GTAA_1C_ criterion as a new definition of PR, based on routine clinical parameters of GV and independent from insulin treatment management, which showed strong correlation with parameters of *β*-cell function. Longitudinal studies are now mandatory for external validation of the potential of GTAA_1C_ to identify PR patients with new-onset T1D.

## Figures and Tables

**Table 1:** Characteristics of the clinical series at diagnosis.

		Total (*n* = 239)	PR (*n* = 170)	No PR (*n* = 69)	*P* ^a^
Gender *n* (%)					0.6
Girls		112 (46.9)	78 (69.6)	34 (30.4)	0.3
	<5 yrs *n* (%)	9 (8)	5 (55.6)	4 (44.4)	
	5–10 yrs *n* (%)	41 (36.6)	32 (78)	9 (22)	
	≥10 yrs *n* (%)	62 (55.4)	41 (66.1)	21 (33.9)	
Boys		127 (53.1)	92 (72.4)	35 (27.6)	0.2
	<5 yrs *n* (%)	30 (23.6)	18 (60)	12 (40)	
	5–10 yrs *n* (%)	47 (37)	36 (76.6)	11 (23.4)	
	≥10 yrs *n* (%)	50 (39.4)	38 (76)	12 (24)	
Age at Δ					0.1
	<5 yrs *n* (%)	39 (16.3)	23 (59)	16 (41)	
	5–10 yrs *n* (%)	88 (36.8)	68 (77.3)	20 (22.7)	
	≥10 yrs *n* (%)	112 (46.9)	79 (70.5)	33 (29.5)	
	Mean (yrs^b^)	9.1 ± 3.8	9.2 ± 3.6	8.8 ± 4.34	
	Median (yrs^c^)	9.7 (6.3–11.9)	9.67 (6.6–11.8)	9.67 (5.3–12.2)	0.7
	Range (yrs)	0.8–16.4	1.8–16.4	0.8–16.2	
	Girls (yrs)^c^	10.3 (7.2–12.3)	10 (7.2–12.2)	11.3 (7.7–12.2)	0.03
	Boys (yrs)^c^	8.9 (5–11.6)	9.4 (5.7–11.5)	7.5 (3.5–11.5)	

^a^Categorical variables were analyzed using chi-square test; ages at diagnosis were analyzed using Mann–Whitney rank sum test (PR-no PR). ^b^Mean ± SD; ^c^median and interquartile range (q25%–q75%); Δ: diagnosis; yrs: years.

## Supplementary Materials

Supplementary Materials Table S1: characteristics of DKA at diagnosis among subgroups of patients with or without PR. Table S2: rates of severe hypoglycemia diagnosis among subgroups of patients with or without PR. Table S3: determinants of IDAA1C-defined PR among glycemic variability parameters. Figure S1: prediction of PR occurrence using the IDAA1C and GVAA_1C_ definitions. Graphs show the percentage of patients that experienced either no PR, PR< 300 days, or PR> 300 days, according to the IDAA1C (a) or the GVAA_1C_ (b) definition.
