# Supplementary material for: Integration of Routine Parameters of Glycemic Variability in a Simple Screening Method for Partial Remission in Children with Type 1 Diabetes
Source: J Diabetes Res. 2018 Jan 17;2018:5936360. doi: 10.1155/2018/5936360 (PMC5822787; doi:10.1155/2018/5936360)
Supplement: Supplementary Materials — Table S1: characteristics of DKA at diagnosis among subgroups of patients with or without PR. Table S2: rates of severe hypoglycemia diagnosis among subgroups of patients with or without PR. Table S3: determinants of IDAA1C-defined PR among glycemic variability parameters. Figure S1: prediction of PR occurrence using the IDAA1C and GVAA1C definitions. Graphs show the percentage of patients that experienced either no PR, PR< 300 days, or PR> 300 days, according to the IDAA1C (a) or the GVAA1C (b) definition. [file 5936360.f1.doc]

**Table S1. Characteristics of DKA at diagnosis among subgroups of patients with or without PR.**

**Table S2. Rates of severe hypoglycemia diagnosis among subgroups of patients with or without PR.**

**Table S3. Determinants of IDAA1C-defined PR among glycemic variability parameters.**

**Figure S1: Prediction of PR occurrence using the IDAA1C and GVAA_1C_ definitions.** Graphs show the percentage of patients that experienced either no PR, PR<300 days or PR>300 days, according to the IDAA1C (**A**) or the GVAA_1C_ (**B**) definition.

**Table S1. Characteristics of DKA at diagnosis among subgroups of patients with or without PR.**

|  | **Total (*n*=214)** | **PR**  **(*n*=154)** | **No PR (*n*=60)** | ***P*^a^** |
| --- | --- | --- | --- | --- |
| **DKA – *n* (%)** | 51 (23.8) | 31 (60.8) | 20 (39.2) | 0.04 |
| **Age at ∆** |  |  |  | 1 |
| <5 yrs (*n*=38) |  |  |  | 0.13 |
| DKA – *n* (%) | 9 (23.7) | 3 (33.3) | 6 (66.7) |  |
| No DKA – *n* (%) | 29 (76.3) | 19 (65.5) | 10 (34.5) |  |
| 5-10 yrs (*n*=73) |  |  |  | 0.49 |
| DKA – *n* (%) | 17 (23.3) | 13 (76.5) | 4 (23.5) |  |
| No DKA – *n* (%) | 56 (76.7) | 47 (83.9) | 9 (16.1) |  |
| ≥10 yrs (*n*=103) |  |  |  | 0.32 |
| DKA – *n* (%) | 25 (24.3) | 15 (60) | 10 (40) |  |
| No DKA – *n* (%) | 78 (75.7) | 57 (73.1) | 21 (26.9) |  |
| **Gender** |  |  |  | 1 |
| **Girls (*n*=101)** |  |  |  | 0.15 |
| DKA – *n* (%) | 24 (23.8) | 14 (58.3) | 10 (41.7) |  |
| No DKA – *n* (%) | 77 (76.2) | 57 (74) | 20 (26) |  |
| **Boys (*n*=113)** |  |  |  | 0.16 |
| DKA – *n* (%) | 27 (23.9) | 17 (63) | 10 (37) |  |
| No DKA – *n* (%) | 86 (76.1) | 66 (76.7) | 20 (23.3) |  |

^a^Categorical variables were analyzed using chi-square test or Fisher exact test according to the sample size; Δ: diagnosis; DKA: diabetic ketoacidosis.

**Table S2. Rates of severe hypoglycemia diagnosis among subgroups of patients with or without PR.**

|  | **Total (n=239)** | **PR (n=170)** | **No PR (n=69)** | ***P*ª** |
| --- | --- | --- | --- | --- |
| **SH** — n (%) | 64 (26.8) | 40 (23.5) | 24 (34.8) | 0.075 |
| **Age at ∆** |  |  |  |  |
| <5 yrs | 39 | 23 (59) | 16 (41) | 0.18 |
| SH — n (%) | 17 (43.6) | 8 (34.8) | 9 (56.3) |  |
| No SH — n (%) | 22 (56.4) | 15 (65.2) | 7 (43.7) |  |
| 5-10 yrs | 88 | 68 (77.3) | 20 (22.7) | 0.025 |
| SH — n (%) | 30 (34.1) | 19 (27.9) | 11 (55) |  |
| No SH — n (%) | 58 (65.9) | 49 (72.1) | 9 (45) |  |
| ≥ 10 yrs | 112 | 79 (70.5) | 33 (29.5) | 0.56 |
| SH — n (%) | 17 (15.2) | 13 (16.5) | 4 (12.1) |  |
| No SH — n (%) | 95 (84.8) | 66 (83.5) | 29 (87.9) |  |
| **Gender** |  |  |  |  |
| Girls | 112 | 78 (69.6) | 34 (30.4) | 0.72 |
| SH — n (%) | 24 (21.4) | 16 (20.5) | 8 (23.5) |  |
| No SH — n (%) | 88 (78.6) | 62 (79.5) | 26 (76.5) |  |
| Boys | 127 | 92 (72.4) | 35 (27.6) | 0.033 |
| SH — n (%) | 40 (31.5) | 24 (26.1) | 16 (45.7) |  |
| No SH — n (%) | 87 (68.5) | 68 (73.9) | 19 (54.3) |  |
| **Mean number of SH** | 2 ± 1.5 | 2.1 ± 1.7 | 1.8 ± 0.9 | 0.29 |

^a^Categorical variables were analyzed using chi-square test or Fisher exact test according to the sample size; Δ: diagnosis; SH: severe hypoglycemia.

**Table S3. Determinants of IDAA1C-defined PR among glycemic variability parameters.**

|  | **Coefficient** | **Standard error** | ***P*** |
| --- | --- | --- | --- |
| Constant | 4.649 | 0.35 | <0.001 |
| HbA_1C_ | 1.032 | 0.0242 | <0.001 |
| % normoglycemia | -3.137 | 0.215 | <0.001 |
| Mean blood glucose | 0.00433 | 0.00127 | <0.001 |
| Standard deviation | 0.00191 | 0.00197 | 0.331 |
| CV | -0.34 | 0.286 | 0.235 |

**Figure S1**
